# Patella Eversion Reduces Early Knee Range of Motion and Muscle Torque Recovery after Total Knee Arthroplasty: Comparison between Minimally Invasive Total Knee Arthroplasty and Conventional Total Knee Arthroplasty

**DOI:** 10.1155/2011/854651

**Published:** 2010-12-29

**Authors:** Tokifumi Majima, Osamu Nishiike, Naohiro Sawaguchi, Kouichi Susuda, Akio Minami

**Affiliations:** ^1^Department of Joint Replacement and Tissue Engineering, Graduate School of Medicine, Hokkaido University, N-15, W-7, Kita-Ku, Sapporo 060-8638, Japan; ^2^Department of Orthopaedic Surgery, Graduate School of Medicine, Hokkaido University, Sapporo 060-8638, Japan; ^3^Shin-Sapporo Orthopaedic Hospital, Sapporo 004-0022, Japan

## Abstract

We hypothesized that patella eversion during total knee arthroplasty (TKA) reduces early return of active knee extension and flexion, quadriceps muscle strength, and postoperative pain. In 100 conventional TKA knees and 100 minimally invasive TKA (MIS TKA) knees, we compared knee range of motion (ROM), postoperative pain, and quadriceps muscle strength at 1 day, 4 days, 1 week, 2 weeks, 3 weeks, 4 weeks, 12 weeks, 1 year, and 5 years after surgery. The differences of surgical approach between MIS TKA and conventional TKA of this study are length of skin incision with subcutaneal flap and patella eversion. In MIS TKA, skin incision is shorter than conventional TKA. Furthermore, patella is not everted in MIS TKA procedure. There were no significant differences in preoperative factors. Postoperative improvement of ROM, postoperative muscle strength recovery, and postoperative improvement of visual analog scale were faster in patients with MIS TKA when compared to that in patients with conventional TKA. On the other hand, no significant difference was observed in complication, 5-year clinical results of subjective knee function score, and the postoperative component angle and lower leg alignment. These results indicate that patella eversion may affect muscle strength recovery and postoperative pain.

## 1. Introduction

Total knee arthroplasty (TKA) is an established treatment for advanced arthritis of the knee [1, 2]. At the time of surgery, medial parapatellar approach, midvastus approach, or subvastus approach is used. The most popular approach of TKA is a medial parapatellar approach that splits quadriceps tendon with eversion of the patella [3]. Having operated without disruption of the extensor mechanism subvastus approach theoretically preserves the quadriceps muscles function. It has been reported that subvastus approach in TKA could regain quadriceps muscle strength faster than the medial parapatellar approach [4–6]. On the other hand, it is reported that performing a knee arthroplasty through a subvastus approach is difficult in patients with limited preoperative knee flexion or in obese patients [7]. Therefore, some surgeons use the midvastus muscle-splitting approach. With the use of this approach, complete eversion and lateral displacement of the patella are not inhibited, allowing for adequate exposure to the joint [8]. In these three approaches, surgeons need an approximately 20 cm length incision of the skin and patella eversion. That may affect length of hospitalization, medical cost, postoperative pain, and length of rehabilitation. 

Minimally invasive surgical techniques have been used in many types of surgical procedures, both arthroscopic surgery and open surgery. Minimally invasive surgery (MIS) as applied to TKA has been developed to improve pain, period of time consumed for functional recovery, blood loss, and hospitalization. Previous reports indicated the advantages of MIS TKA according to 2-year short-term clinical results [9–17]. In the previous study, however, no mid-term more than 5-year clinical results have been reported. Furthermore, no report explained the mechanism of quick pain relief and muscle strength recovery in MIS TKA.

In this paper, we hypothesized that patella eversion will reduce early return of active knee extension and flexion, quadriceps strength, and postoperative pain. To test this hypothesis, we compared knee range of motion (ROM), postoperative pain, and quadriceps muscle strength after TKA in conventional TKA and MIS TKA. The purpose of this study was to evaluate the knee ROM, postoperative pain, and quadriceps muscle strength within one year after surgery. Furthermore, mid-term at least 5-year clinical results of the MIS TKA and conventional TKA were compared. 

## 2. Materials and Methods

Between April 2004 and August 2005, a prospective comparative study was conducted in two hospitals. 200 primary TKAs of 180 patients with medial compartmental osteoarthritic knee were enrolled. Conventional TKA was performed in 100 knees at one hospital. MIS-TKA was performed in 100 knees with age and gender matched to conventional TKA group at the other hospital. All of the patients were evaluated. Follow-up period ranged from 60 to 76 months (average: 66 months). Age at surgery ranged from 60 to 82 years old (average: 70.3 years old). Average preoperative Hospital for Special Surgery scoring system (HSS score) [18] was 46.7 ± 8.5 points in the MIS group and 48.6 ± 7.6 points in the conventional group. No patients received previous surgery on the affected knees. No patients had flexion contracture more than 30 degrees. General anesthesia with epidural tubing was made by the anesthetist. All of the surgery was performed by a single surgeon with subvastus approach using Scorpio NRG PS (Stryker, Mahwah, NJ). The patella was resurfaced in all patients. All of the components were fixed with bone cement.

**Figure 1:**
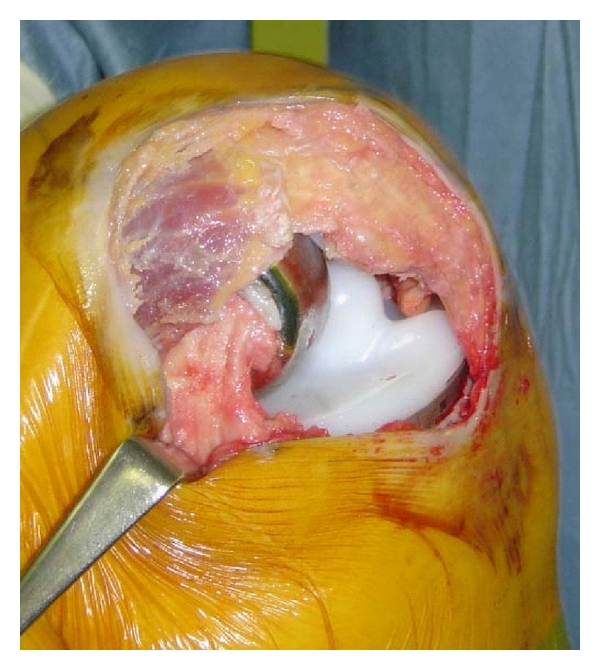
Subvastus arthrotomy with implants is shown from medial side. The vastus medialis oblique muscle and its aponeurosis remain intact and fully attached to the patella.

For the MIS group, after the tourniquet was inflated, a curved medial skin incision extending from the superior pole of the patella to the top of the tibial tubercle was made. Medial arthrotomy was made from the superior pole of the patella to just above the insertion of pes anserinus, and then subperiosteal medial soft tissue release was performed as standard procedure. The patella was displaced laterally without eversion, and first resection of the patella was performed to increase working space. The tibial resection was then performed from the anteromedial side with an extramedullary guide. The distal femur was also resected using an intramedullary guide. The rotation of femoral component was set parallel to the tibial cut surface under individual 40 N load of medial and lateral space using ligament tension with knee flexed at 90 degrees (EndoPlus, Marl, Germany). Further resection of the patella was done to match the thickness of patellar components.  Figure 1 shows subvastus arthrotomy with implant in the MIS TKA group. In the standard group, the surgery was done through a midline incision of about 13 to 18 cm and a medial parapatellar arthrotomy; the patella was everted laterally when the knee was flexed. Tourniquet time was measured in all patients. A suction drain and epidural tube were left for 24 hours in all patients. Postoperative pain management was the same for both groups, which included continuous epidural injection of 2.5 ml/hr of bupivacaine hydrochloride hydrate (Marcain, AstraZeneca, Osaka, Japan) and nonsteroidal anti-inflammatory drugs for 2 weeks. Both groups went through the same clinical pathway with continuous passive motion and bedside physiotherapy started on the second day after surgery. 

Total blood loss and possible time of straight leg raise were measured. Visual analogue scale (VAS) score of pain was scheduled to evaluate at day 1, day 4, 1 week, 2 weeks, 3 weeks, and 4 weeks after surgery in every patient. When responding to a VAS item, patients specify their level of pain to a statement by indicating a position along a continuous horizontal line (100 mm in length) between two endpoints. The VAS score is determined by measuring in millimeters. In VAS score, 0 mm means no pain, and 100 mm means very severe pain. Passive knee ROM was measured using a goniometer. Knee extension muscle torque was recorded at 1 week, 2 weeks, 3 weeks, 4 weeks, 3 months, 12 months, and the final visit. Peak isokinetic torque of the ipsilateral and contralateral quadriceps muscle was measured at 60 degrees/second of angular velocity using the Cybex II dynamometer (Lumex Inc, Ronkonkoma, NY). This muscle contraction involved knee extension. The torque measured in the untreated knee at the preoperative examination was defined as the reference value in each patient, and torque measured at each postoperative period was represented as a ratio (percentage) of the reference value. With regard to radiology, anteroposterior (AP), lateral, and long-leg weight-bearing views were obtained preoperatively and at the final followup. All radiographs were assessed independently by two observers except for evaluation by surgeons with regard to implant position according to the Knee Society TKA roentgenographic evaluation form [19] and mechanical axis of lower leg allowing for an unbiased radiographic assessment.

Statistically, the 2 groups were compared using Student is *t*-test for continuous data. For muscle strength, change in ROM, and change in VAS a two-way analysis of variance with a Bonferroni correction for multiple comparisons was used to analyze the change in time and difference between groups. A *P* value less than  .05 was considered significant.

## 3. Results

The detailed preoperative comparison between the 2 groups is shown in Table 1. There were no significant differences in preoperative factors. 

**Figure 2:**
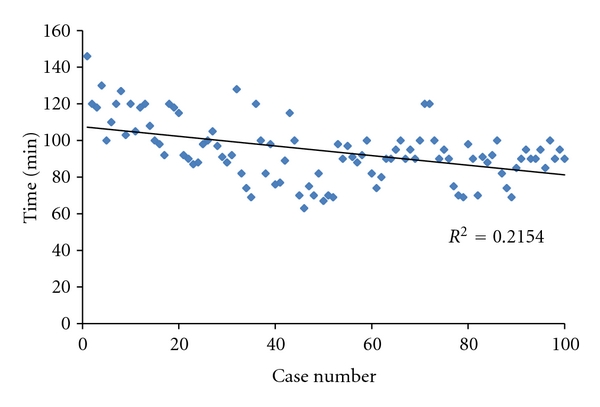
Tourniquet time of 100 cases in MIS-TKA group is shown. Learning curve was observed.

There was no significant difference in the mean tourniquet time (MIS group: 98.9 ± 18.2 minutes; conventional group: 88.6 ± 14.6 minutes). On the other hand, tourniquet time of the early 50 cases was significantly longer than that in the latest 50 cases in MIS-TKA (*P* < .004) (Figure 2). The mean total blood loss also showed no difference between the MIS group (548 ± 348 ml) and the conventional group (631 ± 335 ml). No lateral retinacular release was required in both of the MIS group and the conventional group. Active straight-leg raise was achieved quicker (*P* < .05) in the MIS group (1.02 ± 0.14 days) than in the conventional group (2.13 ± 2.30 days). There were no intraoperative complications in both of the groups. Postoperatively, 5 cases in the MIS group and 2 cases in the conventional group had minor wound complications. There were no deep or superficial infections in either group. There was no symptomatic deep venous thrombosis in all cases. There were no perioperative fatal complications in either group. HSS knee score improved to 89.0 ± 7.5 in the MIS group and 87.3 ± 7.1 in the conventional group (Table 2). 

**Figure 3:**
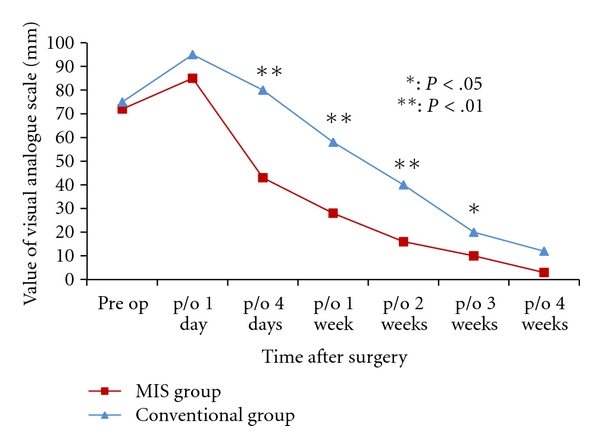
Change of the visual analogue scale (mm) of pain in both groups until 4 weeks after surgery.

**Figure 4:**
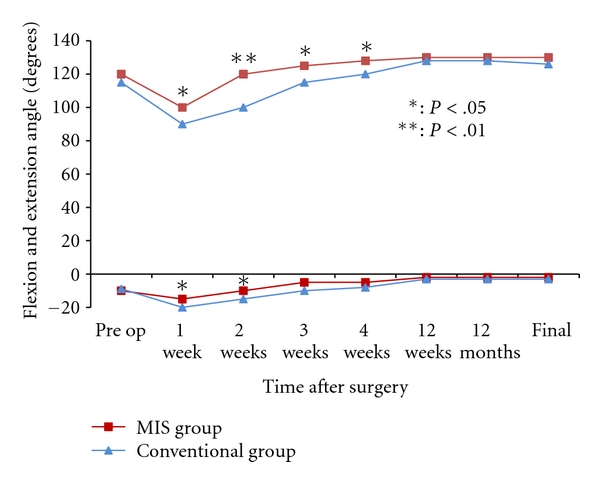
Change in the knee range of motion in both groups until 4 weeks after surgery. Higher range data set represents knee flexion angle. Lower range data set represents knee extension angle.

**Figure 5:**
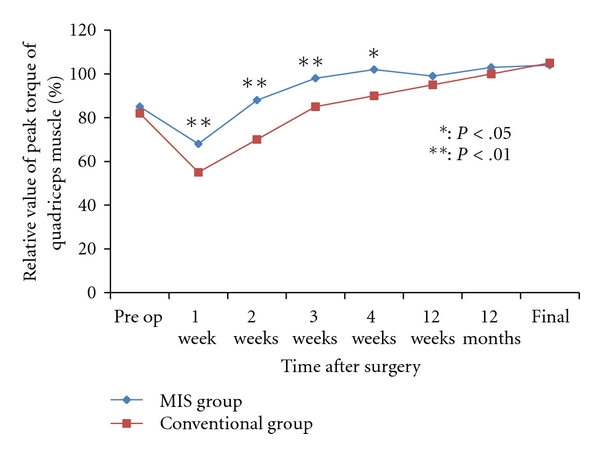
Change in percentage of peak isokinetic torque of the quadriceps muscle in the MIS group and the conventional group is shown. Preoperative peak value in untreated knee was defined as a reference value (100%).

Average postoperative pain between day 4 and 3 weeks after surgery, as recorded on the VAS, was significantly lower in the MIS group (Figure 3). Until 4 weeks after surgery, average knee ROM was larger in the MIS group than in the conventional group (Figure 4). The changes of quadriceps muscle strength in both groups are shown in Figure 5. Peak isometric torque of the quadriceps muscle in the MIS group recovered more quickly than the conventional group. Three months after surgery, there were no significant differences in knee ROM and quadriceps muscle strength.

There were no significant differences in radiological measurements. At the final followup, mechanical axis was 180.4 ± 2.8° in the MIS group and 180.9 ± 2.5° in the conventional group. Cases of overall lower leg alignment of neutral ±3° were 79% and 81% in the MIS group and the conventional group, respectively. Angles of component alpha, beta, gamma, and delta are 94.7 ± 3.6, 88.9 ± 2.0, 90.6 ± 2.7, and 90.3 ± 2.6° in the MIS group and 96.5 ± 1.9, 89.7 ± 2.3, 92.3 ± 2.1, and 91.1 ± 2.3° in the conventional group, respectively. 

## 4. Discussion

In this paper, we hypothesized that patella eversion will deteriorate early return of knee range of extension and flexion, quadriceps strength, and postoperative pain. This paper showed that postoperative improvement of ROM, postoperative muscle strength recovery, and postoperative improvement of pain were greater in patients with MIS TKA when compared to patients with conventional TKA. On the other hand, no significant difference was observed in complication, 5-year mid-term results of knee function score, or the postoperative component angle and lower leg alignment. The strength of the study lies in that we firstly explained the mechanism of pain relief and muscle strength recovery in MIS TKA.

The differences of surgical approach between MIS TKA and conventional TKA of this study are length of skin incision with subcutaneal flap and patella eversion. It has been reported that knee pain with muscle contraction played a small role in the reduction of muscle activation [20]. Concerning the peak torque of quadriceps muscle, traction force to muscle by patellar eversion during surgery may affect the recovery of muscle strength. As for postoperative pain, differences in visual analogue scale of pain exist after wound healing. These results indicate that patella eversion may affect postoperative muscle torque recovery and pain. However, there is possibility that the reduced length of the incision might have contributed to the results. To assess this possibility, we are planning to compare the result of TKA in the same skin incision with patellar eversion and that without patellar eversion for future research.

In the present study, the risks and benefits of MIS TKA and conventional TKA were also compared. The results showed that MIS-TKA is better in postoperative early recovery of muscle strength, while concerning the component placement and lower leg alignment, no deterioration occurred. These results are consistent with previous reports [9–17, 21, 22]. However, the MIS-TKA through a limited field has difficulties. The higher rate of complications such as malalignments, fixation errors, soft tissue damage, increased operating time, and blood loss were also noted [10–13, 15, 23, 24]. Knowing these complications at the time of MIS surgery, we meticulously checked the position of instrument and cut surface to avoid outliers. This effort may result in no case of outliers in alignment, bleeding, and wound healing.

Benefits from an earlier recovery with MIS TKA lead to earlier discharge, reduced physiotherapy, and reduced dosage of pills for pain control. These benefits are convenient for patients. Furthermore, the benefits may reduce medical cost. On the other hand, there were no differences in function and pain 12 weeks after surgery. Three months after TKA, functional recovery and wound healing involving muscle, ligament, and joint capsule may reach plateau whether MIS surgery was performed or not.

Operating through the subvastus approach in MIS TKA was technically demanding for access and overall visibility. Consequently, the tourniquet time was increased by an average of 10 minutes in the initial 50 cases of MIS TKA. On the other hand, this did not influence the incidence of deep venous thrombosis. Despite reduced access in surgical approach, component geometry and leg alignment distributed in acceptable range. We, as a surgical team, have gone through a learning curve and have not experienced intraoperative complications. 

Poor visualization of the knee joint may leads to wrong bone cutting that lead to poor clinical results [25, 26]. Concerning implications of the results, surgeons may extend skin incision without patella eversion when exposure of the knee joint is not enough to visualize the area of bone cutting and soft tissue release.

A limitation of this study was that the patients were not assigned randomly at the same hospital. This fact has a possibility of confounding variables. Another limitation was that 5-year mid-term clinical data with MIS TKA can provide limited information concerning their efficacy and safety. Long-term follow-up studies are necessary. 

In conclusion, MIS TKA is better in early functional recovery of the knee compared to conventional TKA. This effectiveness may owe to the approach without patella eversion.

## Figures and Tables

**Table 1:** Patient demographics.

	Conventional TKA	MIS-TKA	*P* value
Age	69.2 ± 8.1	71.8 ± 7.7	N.S
Sex, F/M	82/18	79/21	N.S
Height (cm)	154.1 ± 6.0	151.5 ± 5.2	N.S
Weight (kg)	60.8 ± 6.1	62.2 ± 5.8	N.S
Preoperative knee extension (degree)	−10 ± 8.5	−8.5 ± 10.1	N.S
Preoperative knee flexion (degree)	115 ± 10.5	120 ± 11.4	N.S
Preoperative mechanical axis in one leg standing radiograph (degree)	190.8 ± 8.9	188.6 ± 10.6	N.S
Percentage of preoperative peak isokinetic torque of the quadriceps muscle (%)*	82 ± 14	85 ± 11	N.S
Preoperative visual analogue scale of pain (mm)	75 ± 11	72 ± 13	N.S
Preoperative knee score	48.6 ± 7.6	46.7 ± 8.5	N.S

*preoperative peak value in uninvolved knee was defined as a reference value (100%).

**Table 2:** Clinical results.

	MIS-TKA	Conventional TKA	*P* value
Mean tourniquet time (min)	98.9 ± 18.2	88.6 ± 14.6	N.S
Total blood loss (ml)	548 ± 348	631 ± 335	N.S
Possible SLR (day)	1.02 ± 0.14	2.13 ± 2.30	*P* < .05
HSS knee score	89.0 ± 7.5	87.3 ± 7.1	N.S
Knee extension (degree)	−2.0 ± 3.1	−3.0 ± 4.1	N.S
Knee flexion (degree)	130 ± 8.1	126 ± 6.5	N.S

SLR: straight leg raise.

## References

[1] Ranawat CS, Flynn WF, Saddler S, Hansraj KK, Maynard MJ (1993). Long-term results of the total condylar knee arthroplasty: a 15-year survivorship study. *Clinical Orthopaedics and Related Research*.

[2] Kelly MA, Clarke HD (2002). Long-term results of posterior cruciate-substituting total knee arthroplasty. *Clinical Orthopaedics and Related Research*.

[3] Insall J (1971). A midline approach to the knee. *Journal of Bone and Joint Surgery A*.

[4] Berth A, Urbach D, Neumann W, Awiszus F (2007). Strength and voluntary activation of quadriceps femoris muscle in total knee arthroplasty with midvastus and subvastus approaches. *Journal of Arthroplasty*.

[5] Roysam GS, Oakley MJ (2001). Subvastus approach for total knee arthroplasty: a prospective, randomized, and observer-blinded trial. *Journal of Arthroplasty*.

[6] Cila E, Güzel V, Özalay M (2002). Subvastus versus medial parapatellar approach in total knee arthroplasty. *Archives of Orthopaedic and Trauma Surgery*.

[7] Knezevich S, Engh GA, Preidis FE (1992). Comparison of subvastus quadriceps-sparing and standard anterior quadriceps-splitting approaches in total and unicompartmental knee arthroplasty. *Orthopaedic Transactions*.

[8] Engh GA, Holt BT, Parks NL (1997). A midvastus muscle-splitting approach for total knee arthroplasty. *Journal of Arthroplasty*.

[9] Tenholder M, Clarke HD, Scuderi GR (2005). Minimal-incision total knee arthroplasty: the early clinical experience. *Clinical Orthopaedics and Related Research*.

[10] Boerger TO, Aglietti P, Mondanelli N, Sensi L (2005). Mini-subvastus versus medial parapatellar approach in total knee arthroplasty. *Clinical Orthopaedics and Related Research*.

[11] Dalury DF, Dennis DA (2005). Mini-incision total knee arthroplasty can increase risk of component malalignment. *Clinical Orthopaedics and Related Research*.

[12] Laskin RS (2004). Minimally invasive total knee replacement using a mini-mid vastus incision technique and results. *Surgical Technology International*.

[13] Tria AJ, Coon TM (2003). Minimal incision total knee arthroplasty early experience. *Clinical Orthopaedics and Related Research*.

[14] Bonutti PM, Neal DJ, Kester MA (2003). Minimal incision total knee arthroplasty using the suspended leg technique. *Orthopedics*.

[15] Haas SB, Cook S, Beksac B (2004). Minimally invasive total knee replacement through a mini midvastus approach: a comparative study. *Clinical Orthopaedics and Related Research*.

[16] Berger RA, Sanders S, Gerlinger T, Della Valle C, Jacobs JJ, Rosenberg AG (2005). Outpatient total knee arthroplasty with a minimally invasive technique. *Journal of Arthroplasty*.

[17] Pagnano MW, Meneghini RM (2006). Minimally invasive total knee arthroplasty with an optimized subvastus approach. *Journal of Arthroplasty*.

[18] Ranawat CS, Insall J, Shine J (1976). Duo condylar knee arthroplasty. Hospital for Special Surgery design. *Clinical Orthopaedics and Related Research*.

[19] Ewald FC (1989). The Knee Society total knee arthroplasty roentgenographic evaluation and scoring system. *Clinical Orthopaedics and Related Research*.

[20] Mizner RL, Petterson SC, Stevens JE, Vandenborne K, Snyder-Mackler L (2005). Early quadriceps strength loss after total knee arthroplasty: the contributions of muscle atrophy and failure of voluntary muscle activation. *Journal of Bone and Joint Surgery A*.

[21] Bonutti PM, Mont MA, Kester MA (2004). Minimally invasive total knee arthroplasty: a 10-feature evolutionary approach. *Orthopedic Clinics of North America*.

[22] Tria AJ (2004). Minimally invasive total knee arthroplasty: the importance of instrumentation. *Orthopedic Clinics of North America*.

[23] Laskin RS, Beksac B, Phongjunakorn A (2004). Minimally invasive total knee replacement through a mini-midvastus incision: an outcome study. *Clinical Orthopaedics and Related Research*.

[24] Chen AF, Alan RK, Redziniak DE, Tria AJ (2006). Quadriceps sparing total knee replacement. The initial experience with results at two to four years. *Journal of Bone and Joint Surgery B*.

[25] Aglietti P, Buzzi R (1988). Posteriorly stabilised total-condylar knee replacement. Three to eight years’ follow-up of 85 knees. *Journal of Bone and Joint Surgery B*.

[26] Jeffery RS, Morris RW, Denham RA (1991). Coronal alignment after total knee replacement. *Journal of Bone and Joint Surgery B*.

